# How *Quercus ilex* L. saplings face combined salt and ozone stress: a transcriptome analysis

**DOI:** 10.1186/s12864-018-5260-2

**Published:** 2018-12-04

**Authors:** Lucia Natali, Alberto Vangelisti, Lucia Guidi, Damiano Remorini, Lorenzo Cotrozzi, Giacomo Lorenzini, Cristina Nali, Elisa Pellegrini, Alice Trivellini, Paolo Vernieri, Marco Landi, Andrea Cavallini, Tommaso Giordani

**Affiliations:** Department of Agriculture, Food and Environment, University of Pisa, Via del Borghetto 80, 56124 Pisa, Italy

**Keywords:** Holm oak, RNA-seq, Transcriptome, Salinity, Ozone, Urban trees

## Abstract

**Background:**

Similar to other urban trees, holm oaks (*Quercus ilex* L.) provide a physiological, ecological and social service in the urban environment, since they remove atmospheric pollution. However, the urban environment has several abiotic factors that negatively influence plant life, which are further exacerbated due to climate change, especially in the Mediterranean area. Among these abiotic factors, increased uptake of Na + and Cl − usually occurs in trees in the urban ecosystem; moreover, an excess of the tropospheric ozone concentration in Mediterranean cities further affects plant growth and survival. Here, we produced and annotated a de novo leaf transcriptome of *Q. ilex* as well as transcripts over- or under-expressed after a single episode of O_3_ (80 nl l-1, 5 h), a salt treatment (150 mM for 15 days) or a combination of these treatments, mimicking a situation that plants commonly face, especially in urban environments.

**Results:**

Salinity dramatically changed the profile of expressed transcripts, while the short O_3_ pulse had less effect on the transcript profile. However, the short O_3_ pulse had a very strong effect in inducing over- or under-expression of some genes in plants coping with soil salinity. Many differentially regulated genes were related to stress sensing and signalling, cell wall remodelling, ROS sensing and scavenging, photosynthesis and to sugar and lipid metabolism. Most differentially expressed transcripts revealed here are in accordance with a previous report on *Q. ilex* at the physiological and biochemical levels, even though the expression profiles were overall more striking than those found at the biochemical and physiological levels.

**Conclusions:**

We produced for the first time a reference transcriptome for *Q. ilex*, and performed gene expression analysis for this species when subjected to salt, ozone and a combination of the two. The comparison of gene expression between the combined salt + ozone treatment and salt or ozone alone showed that even though many differentially expressed genes overlap all treatments, combined stress triggered a unique response in terms of gene expression modification. The obtained results represent a useful tool for studies aiming to investigate the effects of environmental stresses in urban-adapted tree species.

**Electronic supplementary material:**

The online version of this article (10.1186/s12864-018-5260-2) contains supplementary material, which is available to authorized users.

## Background

The woody evergreen sclerophyllous *Quercus ilex* L. (holm oak) is widely distributed in the Mediterranean maquis, extending longitudinally from Portugal to Syria and latitudinally from Morocco to France [1]. This species has been used since the sixteenth century in the landscaping of urban and rural parks [2]. Nowadays, urban trees provide a physiological, ecological and social service in the urban environment [3], since they remove atmospheric pollutants, such as O_3_, NO_2_ and SO_2_ [4], and accumulate airborne particulates [5].

Plants in cities live in a very harsh and constrained environment that involves changes at the morphological and functional levels. In this sense, the concept of urban plant physiology has been developed for assessing how single or multiple environmental factors affect the key environmental services provided by urban forests [6]. The urban environment has many aspects that change over time and interact with each other, such as temperature, light, water availability, soil type, air and soil pollution. Among these abiotic factors, increased uptake of Na^+^ and Cl^−^, the major saline ions, usually occurs in trees in the urban ecosystem, inducing ionic stress that can disturb plant metabolism [7, 8], particularly the photosynthetic process [9]. Moreover, salt stress leads to the alteration of chloroplastic electron flow, which results in the overproduction of reactive oxygen species (ROS) [10]. In response to salt stress conditions, plants exhibit several biochemical and molecular mechanisms to cope with the damaging effects of salinity, such as translocation of Na^+^ from the leaf tissue to vacuoles [7, 11], activation of ion channels, and antioxidant and compatible solute accumulation [12, 13]. Many studies on physiological, molecular, morphological and anatomical adaptation to salt-affected soils in woody plants have recently been reviewed [14]. Concerning *Quercus ilex,* a few articles have described the effect of salinity [15]. In addition, the photosynthetic process and protein profile alterations during drought conditions have been studied [16–20].

Woody sclerophylls are well equipped to face many stresses that often occur simultaneously, especially in the Mediterranean area, such as high irradiance, UV and air pollutants [9, 21]. Anthropic conditions enhance these abiotic factors. For example, the tropospheric O_3_ concentration in Mediterranean cities frequently exceeds the European limit set for the protection of human health and vegetation [22]. This photo-oxidant pollutant influences plant growth, induces an acceleration of leaf senescence, modifications in foliar anatomical characteristics, especially in leaf mass per area and spongy parenchyma thickness. Moreover, O_3_ causes negative effects at both biochemical and physiological levels as a decreasing in the chlorophyll content, and triggers a number of molecular responses in plants, including antioxidant metabolite accumulation and gene expression alterations [23–25].

To date, many studies have been carried out on the effects of different environmental factors occurring simultaneously on growth, yield and physiological traits in plants and crops [26, 27]. Some studies have reported on the combined effects of salinity and O_3_ and were conducted on a long-term basis, where both stressors were supplied simultaneously [28, 29].

In a previous study, Guidi et al. (2017) performed an in-depth physiological and biochemical characterisation of the mechanisms involved in the photosynthetic responses of young saplings of *Q. ilex* subjected to mild salinity stress (150 mM NaCl, 15 days; a realistic dose in the Mediterranean environment) and then subjected to a single pulse of O_3_ (80 nl l^− 1^, 5 h) [30], a situation that plants commonly face, especially in urban environments. Besides the typical response of the plant to salt stress (stomatal closure, reduced CO_2_ photoassimilation and oxidative stress), the results showed that in salt-treated plants an apparently harmful peak of O_3_ required an enhancement of the Halliwell-Asada cycle to counteract the further oxidative load induced by the pollutant [30].

The development of massively parallel sequencing technologies is changing the way by which transcriptomes and genomes are discovered and defined, even in non-model species such as those belonging to the genus *Quercus* [31, 32]. For example, a transcriptome assembly was performed in *Q. pubescens* leaves [33]; Cokus et al. (2015) performed an evolutionary study on California white oaks by assembling a transcriptome after Illumina sequencing of cDNAs from various organs and individuals [34]; Lesur et al. (2015) assembled previously available and newly developed sequence reads of *Q. robur* and *Q. petraea* with the aim of inferring the phylogenetic relationship in the *Quercus* genus and to discover gene networks underlying vegetative bud dormancy release [35]; and recently, Guerrero-Sanchez et al. (2017) compared different assembly methods to obtain a de novo transcriptome assembly from acorn embryo, leaves and roots of *Q. ilex* [36]. In other studies, massively parallel sequencing was also used to evaluate changes in gene expression after stress conditions: Tarkka et al. (2013) produced a reference transcriptome of *Q. robur* to analyse gene expression regulation by a number of biotic stressors (pathogenic and beneficial fungi, nematodes, moths) [37]; Pereira-Leal et al. (2014) produced a comprehensive transcriptome of *Q. suber*, assembling many 454 EST libraries from tissues and organs of plants subjected to *Phytophtora cinnamoni*, mycorrhizal symbiosis and abiotic stresses (drought, salt and oxidative/light) [38]; and recently, Gugger et al. (2017) assessed the whole-transcriptome response to water stress in different genotypes of *Quercus lobata*, a California endemic oak [39].

To our knowledge, no studies of genes whose expression modulation allows plants to adapt to the combination of salinity and O_3_ stresses occurring in the urban environment are available. In the present study, using Illumina cDNA sequencing, we report for the first time on the construction and annotation of a de novo transcriptome of *Q. ilex* leaves, with the aim of exploring the molecular bases of the response of *Q. ilex* to an O_3_ insult occurring in plants coping with soil salinity, as previously physiologically and biochemically assessed by Guidi et al. (2017) [30].

## Methods

### Plant material and experimental design

The plant materials and the experimental design are the same as reported by Guidi et al. (2017) [30]. In brief, three-year-old half-sib saplings of *Q. ilex* grown under field conditions were potted (6.5 L containers) in a growing medium containing a mixture of Einhetserde Topfsubstrat ED 63 standard soil and sand at the end of autumn (December 2014). Potted plants were then maintained under field conditions. One month before the treatments, plants were irrigated daily with a half-strength Hoagland solution. Salt treatments were imposed from 5 to 20 September 2015, and recently developed leaves were marked at the beginning of the treatment. Plants for salt treatment were provided, at 2 days intervals, with an optimal nutrient solution with increasing concentrations of NaCl (0, 25, 50 and 100 mM). At the end of an 8 day acclimation period, a final concentration of 150 mM NaCl was applied until the 15th day. In the same period control plants were supplied with an optimal nutrient solution. After this time, salt-treated and control plants were transferred into four controlled environment fumigation facilities, which were ventilated with charcoal-filtered air (one box for control (C) and one for salt-treated (S) plants) or treated with a single pulse of O_3_ (80 ± 3 nL L^− 1^, 5 h: one box for control plants (O_3_) and one for salt-treated plants (S + O_3_). The O_3_ exposure was carried out from 09:00 to 14:00 h (local time). The entire methodology of O_3_ exposure was performed according to Nali et al. (2004) [40], and further details on experimental conditions are reported in Guidi et al. (2017) [30].

At the end of the fumigation, current year leaves of four individual plants (four biological replicates) grown under each of the experimental conditions (C, S, O_3_, and S + O_3_) were frozen in liquid nitrogen and stored at − 80 °C for subsequent RNA isolation.

### RNA isolation and sequencing

Total RNA was isolated from leaves of four single plants per treatment (C, S, O_3_, S + O_3_), according to a CTAB (hexadecyl trimethyl-ammonium bromide) method modified by Reid et al. (2006) [41]. Leaves (200 mg) homogenised in liquid nitrogen were lysed at 60 °C for 30 min in 1 mL extraction buffer [CTAB 2% *w*/*v*, polyvinylpyrrolidone (PVP) 2% w/v, Tris-HCl 100 mM pH 8, ethylenediaminetetraacetic acid (EDTA) 25 mM, NaCl 2.0 M, Spermidin 0.5 g/L, β-mercaptoethanol 2% *v*/v]. After incubation, the samples were extracted twice with an equal volume of chloroform:isoamyl alchol (24:1), then nucleic acids were precipitated for 30 min at − 80 °C by adding 3 M Na-acetate and cold isopropanol (1:6 *v*/v). Samples were centrifuged at 8000 x *g* for 30 min at 4 °C to pellet RNA. After washing with aqueous ethanol (70% v/v), samples were centrifuged at 8000 x *g* for 10 min at 4 °C, then supernatants were removed and pellets were solubilised in Tris-EDTA buffer. A DNAse I (Roche, Mannheim, Germany) treatment was utilised to completely remove genomic DNA contamination. Finally, RNAs were purified by phenol/chloroform extraction (1:1 *v*/v) and were precipitated following standard procedures.

Sixteen RNA-Seq libraries (four per treatment) were generated using the TruSeq RNASeq Sample Prep kit (Illumina Inc., San Diego, CA, USA). Poly-A RNA was isolated from total RNA and was chemically fragmented. First- and second-strand cDNA syntheses were followed by end repair, and adenosines were added to the 3′ ends. Adapters were ligated to the cDNA, and 200 ± 25 bp fragments were gel purified and enriched by PCR. The libraries were quantified using a Bioanalyzer 2100 (Agilent Technologies, Santa Clara, CA, USA) and run on the Illumina HiSeq2000 (Illumina Inc.) using version 3 reagents.

Paired-end read sequences 125 bp in length were collected, reads are available on SRA with the bioproject accession PRJNA490658. The quality of the reads was checked using FastQC (v. 0.11.5; Babraham Bioinformatics, Cambridge, UK), and the reads were trimmed with Trimmomatic (v. 0.33; [42]), cropping the first 15 bases and the last 10 bases of each read in order to improve the overall quality. Ribosomal contaminant reads were removed using CLC mapping on *Quercus* ribosomal references from NCBI; non-mapped reads were retained.

### De novo transcriptome assembly and annotation

Sequencing reads from leaves obtained from different treatment libraries were collected to build a de novo transcriptome. The transcriptome was assembled employing CLC-BIO Genomic Workbench version 8.0.3 (QIAGEN Aarhus Prismet, Aarhus, Denmark, hereafter called CLC-BIO), which uses the De Brujin graph algorithm. After performing several tests on k-mer size, the best suitable k-mer length value was 26 bp. Apart from k-mer size, default parameters were used for the assembly. A sequence length cutoff was set as 300 bp, although contigs shorter than this threshold length were also collected when produced by assembling more than 200 reads. To improve transcriptome quality, contigs showing a similarity cluster of over 95% were trimmed using CD-HIT-EST [43].

Contigs were annotated using NCBI Blastx and RefSeq plant database of NCBI [44]. NCBI Blastx ran with the following parameters: maximum number of hits = 10, E-value cutoff = 10^− 5^. Contigs were also analysed on reference *Quercus* spp. transcripts OCV3_91K [35] using Blastn by default parameters.

Gene Ontology (GO) terms, InterPro ID and KEGG ID on annotated contigs were found using Blast2GO [45] with default parameters. GO enrichment analysis with Fisher exact tests on differentially expressed transcripts was performed with Blast2GO analysis tools using *P*-values corrected with a false discovery rate of (FDR) < 0.05; enriched GO terms were summarised using REVIGO with “tiny allowed similarity” parameter [46]. GO-Slim was run to reduce complexity of GO terms for gene class analysis.

### Differential gene expression analysis

Expression abundance of the transcripts was estimated by mapping reads from the individual library onto the de novo transcriptome in the four growth conditions using CLC-BIO. This software counts unique reads and discards multi-reads, or distributes multi-reads at similar loci in proportion to the number of unique reads recorded. In the first case, the expression of genes that have closely related paralogues would be underestimated. Hence, besides unique reads, reads that occurred up to ten times were also included in the analyses, a strategy that should also allow correct estimation of activity for paralogue genes [47].

Raw counts of mapped reads were analysed using the R statistical package edgeR [48]. Gene expression level was calculated as reads per kilobase per million reads mapped (RPKM) as described in Mortazavi et al. (2008) [47]. We filtered out contigs with RPKM < 1 in at least one library.

Aligned reads counts of four replicates of each treatment were analysed with the R statistical package EdgeR [48] as specified by manual instruction and by Anders et al. (2013) [49]. A pairwise comparison test was performed between stressed and control libraries. The resulting *P*-values were corrected with the FDR [50], and contigs showing an FDR corrected *P* value < 0.05 were selected as significant. The fold changes between controls and treatments were considered significant when the expression value of a sample was at least two fold higher or lower than the other samples, splitting contigs into two groups: up-regulated or down-regulated.

A GO term was derived for each contig, slimmed using Blast2GO (plant GO slim) and classified according to biological process, molecular function and cellular component.

## Results

### cDNA sequencing and production of a de novo transcriptome of *Quercus ilex*

For the first time a de novo transcriptome for *Q. ilex* leaves subjected to mild salinity and/or O_3_ treatments was produced and annotated. A total of 543,086,098 sequence reads were generated, each 125 nt in length. The total number of tags per library (independent of the treatment) ranged from 24.96 to 58.75 million (Table 1), a tag density sufficient to assess a de novo transcriptome and for quantitative analysis of gene expression [51]. Filtering reads for quality resulted in a total of 520,727,296 trimmed reads, 100 nt in length, corresponding to a dataset of about 65 Gb of sequence data (Table 1). De novo assembly of high quality reads was performed, and 182,985 contigs were produced. N50 and N75 were 724 nt and 372 nt, respectively. Both indexes were in the range of those previously reported for other transcriptome assemblies of oak tree species [32, 35, 36]. The minimum length of the assembled contigs was 82 nt, the maximum was 15,621 nt, with an average length of 574 nt. The resulting *Q. ilex* leaf transcriptome of control (C), salt treated (S), ozonated (O_3_) and salt plus O_3_ treated (S + O_3_) plants included 126,369 putative transcripts.

**Table 1:** Summary of RNA sequencing and mapping results. Number of raw and trimmed Illumina reads used in the experiments and number of reads matching the de novo *Quercus ilex* leaf transcriptome for each library

Library nr. (and treatment)	Number of raw reads	Number of reads after trimming	Number of mapped reads on *de-novo* transcriptome	% of mapped reads on *de-novo* transcriptome
1 (C)	29,780,158	28,507,766	23,116,305	81.09%
2 (C)	34,707,140	33,397,650	27,251,484	81.60%
3 (C)	30,142,604	28,938,768	23,290,009	80.48%
4 (C)	31,504,924	30,332,808	24,980,007	82.35%
5 (S)	27,286,188	25,418,352	20,578,777	80.96%
6 (S)	28,946,286	27,599,316	22,254,839	80.64%
7 (S)	24,962,794	23,641,096	18,818,381	79.60%
8 (S)	28,231,268	27,499,364	21,995,959	79.99%
9 (O_3_)	27,653,576	26,359,594	21,539,430	81.71%
10 (O_3_)	38,657,480	37,427,844	30,572,382	81.68%
11 (O_3_)	58,745,972	57,078,984	46,381,516	81.26%
12 (O_3_)	35,218,784	34,468,334	28,082,724	81.47%
13 (S + O_3_)	57,199,362	54,995,868	44,442,489	80.81%
14 (S + O_3_)	32,727,622	31,290,440	25,418,644	81.23%
15 (S + O_3_)	27,994,188	26,349,048	21,210,329	80.50%
16 (S + O_3_)	29,327,752	27,422,064	22,169,515	80.85%

*C* Control plants, *S* Salt-treated plants, *O*_*3*_ Ozonated plants, *S + O*_*3*_ Salt plus O_3_ treated plants

In order to confirm the robustness of our assembly a comparison with transcriptomes of *Q. robur* and *Q. petraea* [34] was performed. Blast analyses are reported in Additional file 1: Table S1 and showed that 75,543 contigs were shared with sequences from transcriptomes generated from these close oak species, a number quite similar to that obtained by Guerrero-Sanchez et al. (2017) [36].

After mapping reads of each library using this de novo transcriptome as reference, the percentage of mapped reads ranged from 79.60 to 81.71% (Table 1).

**Fig. 1:**
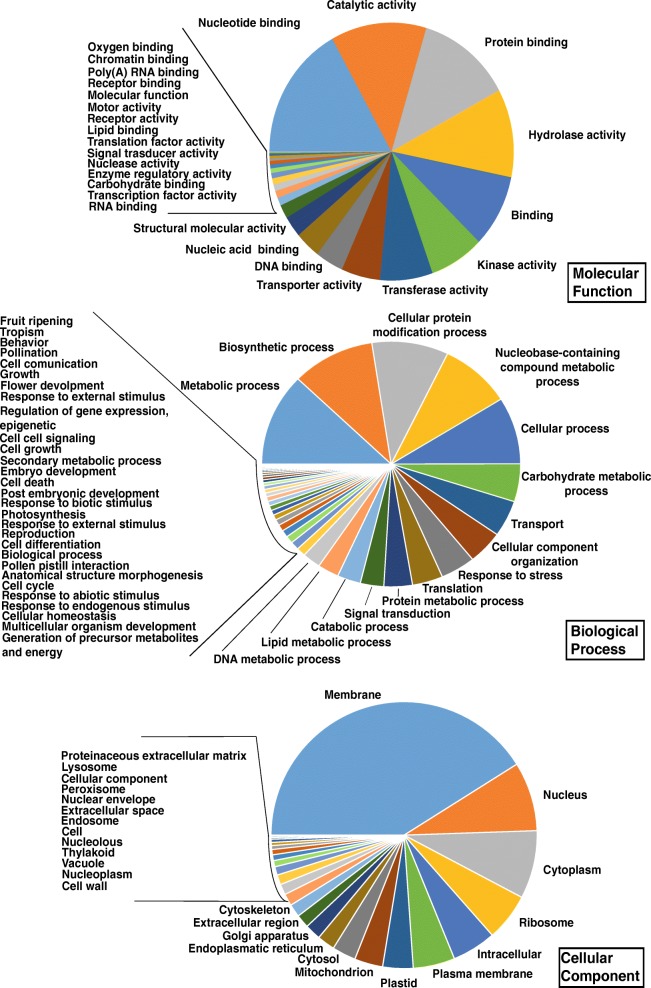
Distributions of GO terms in the transcriptome of *Quercus ilex* leaves. GO terms were subdivided according to three main ontologies: molecular function, biological process, and cellular component

Functional annotation of *Q. ilex* leaf transcriptome sequences was based on sequence alignments to the RefSeq plant database. The total number of annotated contigs amounted to 53,500 (42.3% of whole transcriptome). At least one GO term was attributed to 39,954 contigs. The distribution of GO terms in the transcriptome is reported in Fig. 1, keeping the main ontologies separated: molecular function, biological process, and cellular component. Independently of treatment, the GOs most represented were nucleotide binding (7028/41,434 16.9%), metabolic process (3859/3,1631 12.2%) and membrane (7046/17,152 41.07%) for molecular function, biological process and cellular component, respectively.

### Global analysis of salinity-, ozone- and salinity+ozone-regulated transcripts

A total of 126,369 putative transcripts (contigs), included in the de novo *Q. ilex* leaf transcriptome in the four treatments were evaluated. The analysis was limited to transcripts with RPKM > 1 in at least one of the four individuals in at least one treatment. By this method, we selected 84,264 significantly expressed contigs.

**Fig. 2:**
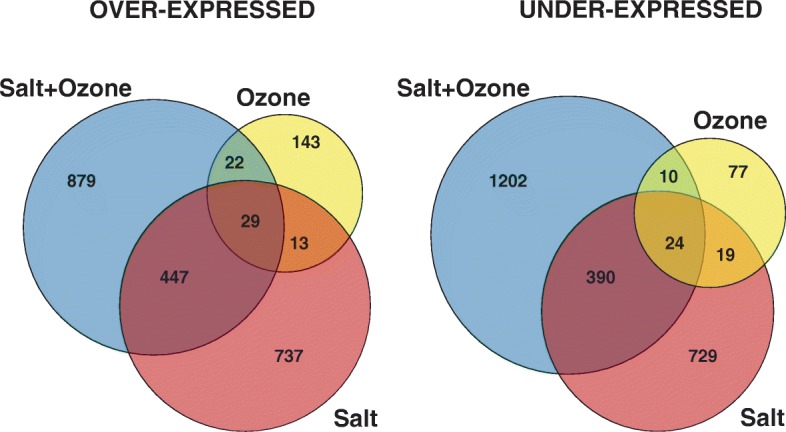
Venn diagrams of over- and under-expressed transcripts in *Quercus ilex* plants treated with salt, ozone, or salt plus ozone compared to control plants

Figure 2 reports the number of transcripts that was significantly over- or under-expressed in S, O_3_ and S + O_3_ plants in comparison to control plants. Overall 2388, 337 and 3003 differentially expressed transcripts were detected in S, O_3_ and S + O_3_ plants, respectively. A comprehensive list of these transcripts is reported in Additional file 2: Table S2, Additional file 3: Table S3, Additional file 4: Table S4. Moreover, a large number of transcripts were specifically (i.e., not shared with other treatments) regulated by salinity and by combined S + O_3_ treatments.

**Fig. 3:**
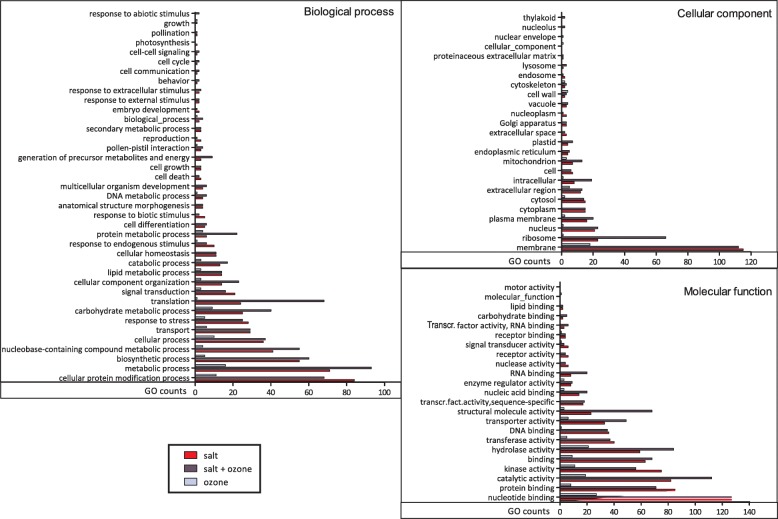
Functional classification of over-expressed transcripts in leaves of *Quercus ilex* plants treated with salt (red bar), O_3_ (light blue bar), and salt plus O_3_ (dark blue bar) compared to control plants. GO terms were subdivided according to their three main ontologies: biological process, cellular component, and molecular function

Based on GO-slim annotations, up- and down-regulated transcripts were classified into three ontological categories: cellular component, biological process, and molecular function. The GO terms of over-expressed transcripts are reported in Fig. 3. A larger number of over-expressed GO terms was identified in S and in S + O_3_ than in O_3_ plants. In S and S + O_3_ plants, 34 and 38 GO terms, respectively, were recognised. Within the biological process category, the most frequent GO terms in S and S + O_3_ plants were cellular protein modification processes, metabolic and biosynthetic processes. Within cellular component, the most frequent GO terms were membrane, ribosome and nucleus, while for molecular function, the most abundant GO terms were nucleotide binding, protein binding, catalytic activity and, in S + O_3_ plants, hydrolase activity (Fig. 3).

For some GO terms, the number of transcripts in S + O_3_ plants was more than two fold compared to S plants, indicating that the short O_3_ pulse affected transcription much more in combination with salt stress than alone. Even though for some GO terms the effect of a single pulse of O_3_ overlapped with those of S and S + O_3_, the distribution of GO terms in O_3_ plants was different from that of S and S + O_3_ plants, especially for the cellular component category. The most represented GO terms (with the exception of membrane) were different between O_3_ and S plants, and the GO term cell wall was even more represented in O_3_ than in S or S + O_3_ plants (Fig. 3). Moreover, for some terms, the effect of O_3_ was almost negligible or even absent (see for example nucleus and ribose for cellular component; RNA and DNA binding for molecular function; and cellular homeostasis for biological process) (Fig. 3).

**Fig. 4:**
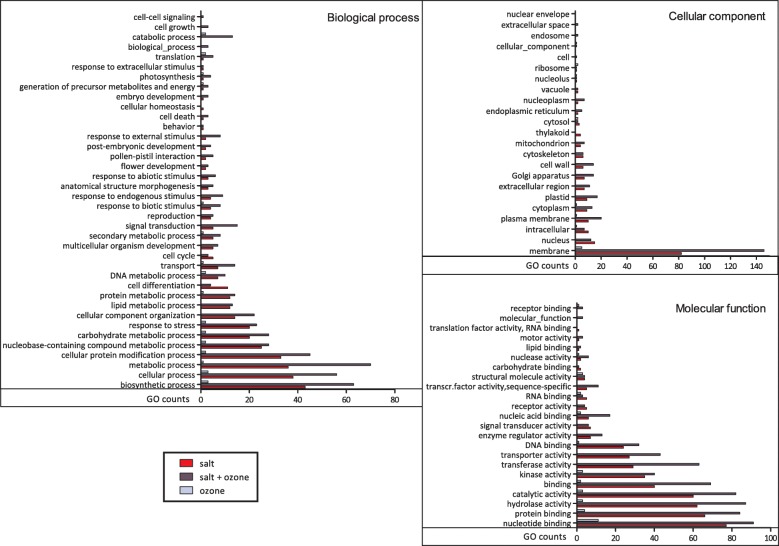
Functional classification of under-expressed transcripts in *Quercus ilex* leaves of plants treated with salt (red bar), O_3_ (light blue bar), and salt plus O_3_ (dark blue bar) compared to control plants. GO terms were subdivided according to their three main ontologies: biological process, cellular component, and molecular function

S and S + O_3_ plants showed also a much higher number of under-expressed GO terms compared to plants treated with O_3_ (Fig. 4). Considering the three main ontological categories, the most represented GO terms were often the same for under-expressed and over-expressed transcripts. For most GO terms, the number of down-regulated transcripts of S + O_3_ plants was much higher than that of S plants (Fig. 4), indicating that a brief O_3_ pulse can dramatically change gene expression when occurring in plants already coping with another abiotic stressor.

### Differentially expressed genes after salt treatment

**Fig. 5:**
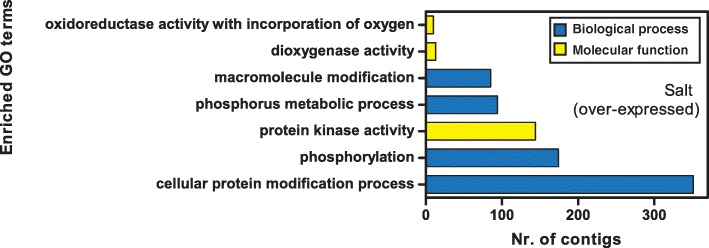
Enriched GO terms in contigs over-expressed in leaves of *Quercus ilex* salt-treated plants

Gene Ontology enrichment analysis showed that significantly enriched GO terms occurred only in the up-regulated gene set (Fig. 5). Among biological processes, the most enriched terms were cellular protein modification and phosphorylation, whereas among molecular functions, the most enriched term was protein kinase activity (Fig. 5). No cellular component term was enriched in salt-regulated transcripts.

The list of differentially expressed transcripts under salt treatment is reported in Additional file 2: Table S2. In total, 1158 of 2389 contigs were annotated.

A comprehensive view of activated transcripts shows that many are involved in stress signalling and scavenging. Concerning the first group some transcripts encode receptors such as G-type- and mitogen activated protein-kinases and other are involved in calcium signalling, for example those encoding calcineurin B-like (CBL) interacting kinases, Ca^++^-binding CML44-like protein and calcineurin subunit B. Also many transcripts encoding transcription factors belonging to both ABA-dependent (MYB, NAC, WRKY) and -independent (ABI, ERF) pathways were found. Other salt activated transcripts were related to other phytohormones such as jasmonic acid, salicylic acid and ethylene. Examples are transcripts encoding linoleate 13S-lipoxygenase 2- chloroplastic-like, 12-oxophytodienoate reductase 3-like, salicylate carboxymethyltransferase-like, DMR6-LIKE oxygenase 2, 1-aminocyclopropane carboxylate oxidase.

Concerning up-regulated sequences related to salt stress scavenging, some are involved in osmolytes biosynthesis (encoding inositol-transporter, trehalose phosphate phosphatase C, phosphatidylinositol phosphatidylcholine transferase). Moreover also sequences encoding ions transporters (plasma membrane calcium-transporting ATPase, potassium transporters, copper transporters), V-type proton ATPase and channels (an aquaporin PIP1–2) were over-expressed.

Other salt activated transcripts were related to photosynthesis, cell wall formation, ROS degradation, and membrane repair (Additional file 2: Table S2).

Many under-expressed transcripts encode members of the ABC transporter family, while others are involved in ABA biosynthesis or in cell wall sugar and protein modifications (bifunctional UDP-glucose 4-epimerase and UDP-xylose 4-epimerase 1-like isoform X4, polygalacturonase, rhamnogalacturonate lyase B, pectate lyase, xylan alpha-glucuronosyltransferase 2 isoform X1, cellulose synthase, pectin acetylesterase 8-like, lysosomal beta glucosidase-like and subtilisin-like protease). Also, a few transcripts involved in ascorbate homeostasis (for example, encoding L-ascorbate peroxidase 6 isoform X2) were down regulated (Additional file 2: Table S2).

### Differentially expressed genes after ozone treatment

As shown in Fig. 2, 207 *Q. ilex* transcripts were significantly over-expressed and 130 were under-expressed by greater than 2-fold following O_3_ treatment when compared to C plants. No GO terms exhibited significant enrichment in the O_3_ up-regulated and down-regulated transcripts.

There were 337 transcript sequences significantly regulated by the O_3_ pulse and these are reported in Additional file 3: Table S3. Among these, 149 were annotated.

An overview of up-regulated transcripts showed that they were mainly involved in stress signalling and ROS scavenging, in particular in calcium signalling, ethylene and auxin network, ROS sensing and degradation. A calcineurin subunit B, a calcium sensor belonging to CBL-interacting kinase family, and a calcium-binding calmodulin 42-like protein encoding transcripts were among the most over-expressed in O_3_ plants. Also two auxin-induced transcripts and transcripts involved in ethylene biosynthesis or in the ethylene network such as those encoding an amino-cyclopropane-1-carboxylate oxidase, an S-adenosyl-methionine decarboxylase, methylenetetrahydrofolate reductase 2-like and an ethylene-responsive transcription factor ERF084 were up-regulated.

Concerning ROS scavenging, transcripts over-expressed by an O_3_ pulse encoded a catalase isozyme and other enzymes related to the Halliwell-Asada cycle, such as mono-dehydro-ascorbate reductase and L-ascorbate peroxidase cytosolic.

A number of transcripts related to cell wall sugar and protein turn-over were found to be over-expressed after O_3_ treatment such as those encoding pectinesterase, beta-galactosidase, polygalacturonase, exopolygalacturonase, bi-functional UDP-glucose 4-epimerase and UDP-xylose 4-epimerase, pectate lyase and subtilisin-like protease.

Other over-expressed transcripts are related to energy metabolism for example encoding ATP synthase subunit mitochondrial, cytochrome c oxidase subunit 5b – mitochondrial-like, V-type proton ATPase subunit a3 and ATP-citrate synthase alpha chain 2-like.

A few transcripts encoding Rubisco interacting proteins and disease related proteins were under-expressed (Additional file 3: Table S3).

### Differentially expressed genes after combined salt and ozone treatment

The gene set regulated by combined salt and O_3_ treatment was the most conspicuous, amounting to 1377 and 1626 over- and under-expressed transcripts, respectively (Fig. 2). Among these, a total of 2081 transcripts (879 over- and 1202 under-expressed) were specifically differentially expressed in combined stress, while 498 over- and 424 under-expressed transcripts were shared between the salt and/or ozone treatments, suggesting that combined treatments have a stronger effect on gene expression modification compared to separate treatments.

**Fig. 6:**
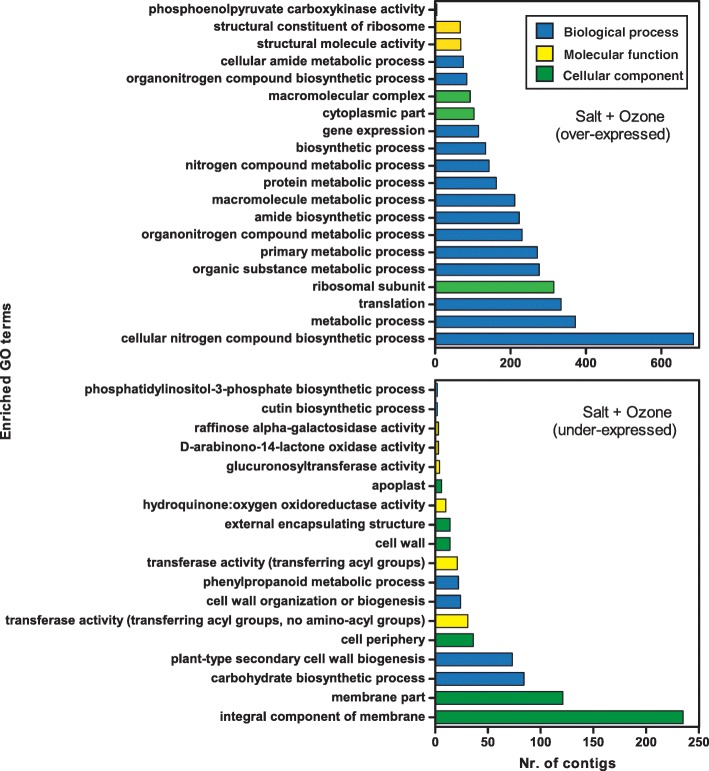
Enriched GO terms in contigs over- or under-expressed in *Quercus ilex* leaves of salt plus O_3_ treated plants

Gene Ontology analysis (Fig. 6) revealed the occurrence of numerous enriched GO terms both in activated and repressed transcripts. Amongst up-regulated gene sequences, the most represented GO categories were cellular nitrogen compound biosynthetic process, metabolic process and translation, all belonging to the general category of biological process. The most enriched cellular component term was ribosomal subunit and the most enriched molecular function term was structural constituent of ribosome. Interestingly, no enriched GO categories were shared between S and S + O_3_ treatments (compare Fig. 5 to Fig. 6), further showing the specificity of the response to the combined stress compared to salt or O_3_ stresses separately.

**Fig. 7:**
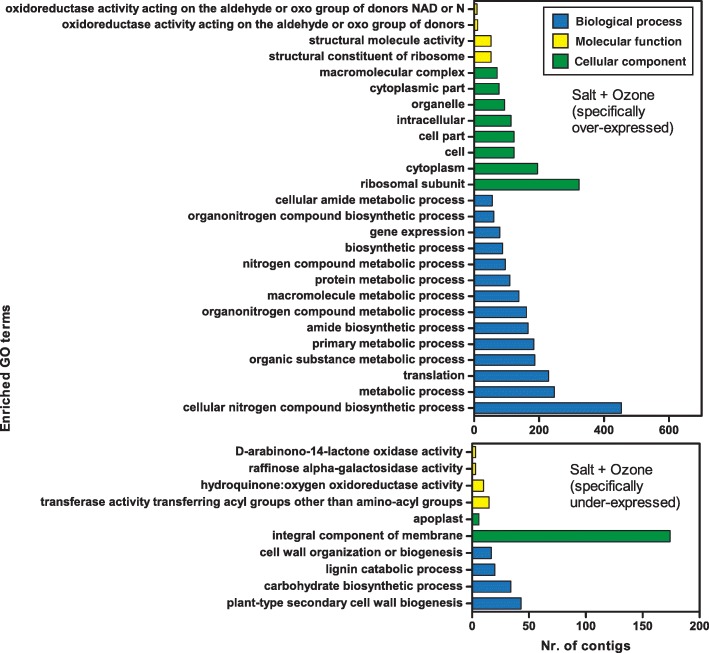
Enriched GO terms in contigs specifically (i.e. not shared with other treatments) over- or under-expressed in *Quercus ilex* leaves of salt plus O_3_ treated plants

In order to explore the biological processes, cellular components, and molecular functions specifically related to the combined S + O_3_ treatment, a GO enrichment analysis was carried out focusing only on the 879 and 1202 transcripts specifically over- and under-expressed in this treatment, respectively (see Fig. 2). For these transcripts, specific roles in response to the combination of stress may be argued. The GO terms specifically affected by the S + O_3_ treatment are reported in Fig. 7.

In total, 26 and 10 GO terms were enriched in the specific over- and under-expressed transcript sets, respectively. Concerning the activated transcripts, the most striking differences between specific (i.e., up-regulated only by S + O_3_, Fig. 7 above) and non-specific (i.e., up-regulated by S + O_3_ and by S and/or O_3_ separately, Fig. 6 above) GO terms were related to cellular component, i.e., cytoplasm, cell, cell part, intracellular and organelle, suggesting that deep changes in the localisation of biochemical activities occurred following the combined stress.

The distribution of GO categories for transcripts specifically repressed by the combined treatment (Fig. 7 below) was different from that for transcripts under-expressed by S + O_3_ and shared with S and/or O_3_ (Fig. 6 below). For example, GO terms such as membrane part, cell periphery, phenylpropanoid metabolic process, transferase activity (transferring acyl groups) were not enriched in the specifically repressed gene set. On the other hand, lignin catabolic process was enriched only in the specifically repressed transcripts.

There were 3003 over- or under-expressed transcripts after combined S + O_3_ treatment and these are reported in Additional file 4: Table S4. Among these, 1488 were annotated. When differentially expressed transcripts in S + O_3_ plants were shared with S or O_3_ plants, the resulting fold change was generally comparable (Additional file 2: Table S2, Additional file 3: Table S3, Additional file 4, Table S4). Among these transcripts there were some involved in signalling such as encoding kinases (CBL-interacting kinase 5-like, cysteine-rich receptor 15, Leucine-rich repeat family isoform 1, LRR receptor-like serine threonine EFR), and other involved in ethylene biosynthesis such as aminocyclopropane-1-carboxylate oxidase. Others included genes related to ROS production or detoxification (catalase isozyme 1) or genes involved in different pathways such as energy metabolism and defence response like those encoding ATP synthase subunit mitochondrial and disease resistance RPM1 isoform X2.

In some shared transcripts a different expression level was also found between individual and combined stress condition responses. Among these, sequences encoding subunit beta of chloroplastic Chaperonin 60 were down-regulated in the O_3_ and S treatments but were unchanged in the S + O_3_ treatment and a probable disease resistance RPP8 2 transcript was highly expressed in O_3_ and S plants but only slightly activated after S + O_3_ treatment. In a few cases, individual and combined stressed plants also showed some contrasting molecular responses. For example, a transcript encoding disease resistance RGA2-like protein was over-expressed in both O_3_ and S plants but were repressed after combined treatment. Similarly, a sequence related to a probable WRKY transcription factor 40 was up-regulated in S plants but was down-regulated in S + O_3_ plants and was unchanged after ozone treatment (Additional file 2: Table S2, Additional file 3: Table S3, Additional file 4, Table S4).

Many transcripts differentially expressed after S + O_3_ treatment were specific to this combined stress (Fig. 2 and Additional file 4: Table S4). An overview of these transcripts indicates they are mainly involved in stress signalling, osmotic adjustments, ROS scavenging and signalling, primary metabolism and cell wall remodelling. Concerning stress signalling, sequences encoding CBL-interacting serine threonine- kinase 25-like, a calcium-binding CML39-like and one specific calcineurin B4-like were down-regulated only in the combined treatment. Concerning transcript putatively involved in osmotic adjustments, one transcript encoding a putative sucrose-phosphate synthase, a transcript encoding a putative inositol transporter 2 and a D-xylose-proton symporter were over-expressed in S + O_3_ stressed holm oaks while a down-regulation of a gene sequence encoding an alpha-trehalose-phosphate synthase was found.

Also, sequences encoding one Mn-SOD, two catalases, three members of the glutathione transferase gene family, a transketolase-chloroplastic, a glyoxylate hydroxypyruvate reductase, as well as several transcripts related to disease resistance were specifically up-regulated in the combined stress. Under-expressed transcripts from S + O_3_ plants encoded a calcium-binding CML39-like protein, an alpha-trehalose-phosphate synthase, and proteins involved in cell wall remodelling such as beta-D-xylosidase 1, fasciclin-like arabinogalactan 11, beta-1,4-xylosyltransferase IRX10, UDP-glucuronate:xylan alpha-glucuronosyltransferase 1-like, cellulose synthase and pectinesterase (Additional file 4: Table S4).

## Discussion

Plants in cities must face multiple environmental factors, which limit their growth and development. Among these factors, salinity and ozone excess are commonly experienced in Mediterranean areas [52]. Here, we analysed transcriptome changes in *Quercus ilex* half-sib saplings after salt, ozone and a combination of salt and ozone treatments by Illumina RNA sequencing.

Oak saplings were grown in salinity conditions for more than 15 days (i.e., simulating long-term stress) in combination with a short (5 h) O_3_ treatment, simulating a condition to which urban plants are often exposed during the day.

Differential expression analyses showed that salt treatment produced greater gene expression alterations than the ozone treatment. This difference is likely related to the different intensities of the two stress treatments. Indeed, compared to the O_3_ treatment, salinity was imposed for a longer period (15 days) to saplings, and the responses to these stresses were detected at the end of both treatments.

Although the O_3_ treatment induced a low number of differentially expressed transcripts between treated and control plants, it was apparent that the short O_3_ pulse had a very strong effect in inducing gene over- or under-expression when it was combined with salt treatment. In fact, the group of over- or under-expressed transcripts in the leaves of plants subjected to the combined S + O_3_ treatment was the most represented (Fig. 2).

These results were confirmed by the analysis of Gene Ontology. Many GO categories of differentially regulated genes occurred in all treatments (S, O_3_ and S + O_3_). The GO counts in O_3_ plants were generally very low, and those of plants subjected to the combined treatment was often much higher than the GO counts regulated by the treatment with salt only (Figs. 3 and 4). Such a great increase of gene regulation determined by a combination of stressors has been observed in other studies; for example, in *Triticum durum* plants subjected to heat and drought separately or in combination [53].

Many differentially expressed transcripts, shared among treatments or treatment specific, will be discussed hereafter in relation to the treatment, modulation, functional role and to biochemical data described by Guidi et al. (2017) [30].

### Differential gene expression under salt treatment

As indicated by Gene Ontology enrichment analysis, salt treatment induced the over-expression of many transcripts encoding kinases, similar to that observed in rice defence mechanisms and stress signalling [54]. Interestingly, among these, a transcript encoding a putative γ**-**subunit PV42a of an SNF1-related kinase was over-expressed. This kinase activates catabolic processes and represses energy consuming anabolic processes and growth [55], suggesting a shift to the remobilisation of allocated carbon skeleton instead of a de novo biosynthesis via anabolic processes.

Calcium is a second messenger for stress responses and plays an important role in salt signalling mechanisms [12]. Many transcripts involved in calcium signalling and perception were over-expressed in leaves of salt-treated holm oak plants.

As observed in other studies [56], in our experiments, salt receptor activation and signalling determined a number of cascade molecular modifications leading to over-expression of both ABA-dependent and -independent transcription factor encoding sequences. However, three transcripts involved in ABA biosynthesis (ABA hydroxylase, phytoene synthase and β-carotene isomerase) were down-regulated, in agreement with previous biochemical data showing no ABA accumulation in salt-treated leaves of *Q. ilex* [30].

In addition, our analysis confirmed that other phytohormones such as jasmonic acid and salicilic acid were involved in plant responses to salinity [57, 58]. Many transcripts related to jasmonic acid and salicylic acid biosynthesis were indeed over-expressed in holm oak leaves grown under salinity. Some of these transcripts have been related to defence-associate responses as a sequence encoding a DMR6-LIKE oxygenase 2 suggesting common regulation pathways between salinity and pathogen responses [59].

Osmolytes are usually accumulated in response to salt stress for salt scavenging [60]. In our previous study, no significant changes in the level of proline produced or in the activity of enzymes related to its metabolism were found [30]. The present transcriptome analysis is in accordance with these data. It is noteworthy that gene sequences related to the biosynthesis of other osmolytes (inositol and trehalose) were up-regulated. Also sequences encoding ions transporters were over-expressed, suggesting that osmotic adjustments in salt-stressed holm oak plants are also controlled at transcriptional level. In particular, a transcript encodes a V-type proton ATPase that exerts a key role in salt tolerance by promoting secondary active Na^+^/H^+^ antiport through the tonoplast [61], ensuring active transport of Na^+^ into the vacuole thus avoiding its harmfulness for the cytosolic compartment. Concerning other transcripts presumed to play a role in salt stress scavenging, one encoding Late Embryogenesis Abundant proteins was over-expressed, as reported by Amara et al. (2014) [62].

Our previous work showed that, in salt-treated oak trees, the photosynthetic process was limited, mainly because of low chloroplast CO_2_ concentration determined by both low stomatal and mesophyll conductance [30]. The CO_2_ assimilation decrease was compensated by a higher efficiency of carboxylative activity of Ribulose-1,5-*bis*phosphate carboxylase/oxygenase (Rubisco). This was found to be coupled with an enhancement of thermal dissipation in the PSII antennae of excess excitation energy in order to avoid possible photodamage to PSII [63]. Most photosynthesis-related transcripts were not affected by salt treatment; however, a gene encoding Rubisco small subunit was slightly induced, while the down-regulation of a transcript encoding a protein involved in Rubisco folding was observed. Interestingly, the amount of transcripts encoding a peroxisomal (S)-2-hydroxy-acid oxidase GLO4 increased with salt treatment. This is a photorespiratory enzyme that can exert a strong regulation of photosynthesis, possibly through a feed-back inhibition on Rubisco activase [64].

Evergreen sclerophylls such as *Q. ilex*, with their long-lived leaves have a low photosynthetic efficiency on a mass basis because these species invest preferentially in vascular and cell wall formation [65]. This induces these species to decrease intercellular spaces and increase cell wall thickness, increasing CO_2_ drawdown but also maintaining high foliar relative water content [66] and osmotic stress tolerance. Concerning genes related to cell wall, salt treatment led to a down-regulation of transcripts encoding proteins involved in modifications of sugars and proteins. Similar results were obtained in *Populus x canescens* under salt stress, in which genes involved in cellulose synthesis were repressed, leading to an increase in the ratio of lignin to cellulose [67]. Interestingly, other transcripts encoding expansins, xyloglucan endotransglucosylase hydrolase and xyloglucan galactosyltransferase were over-expressed in our experiments. These enzymes catalyse the splitting and/or reconnection of xyloglucan cross-links in the cellulose-hemicellulose framework of cell wall, hence they are likely involved in salt-elicited leaf succulence in higher plants [68]. In our experiments, their over-expression in salt-treated plants is likely related to the increased leaf succulence observed in the previous study [30].

Reactive oxygen species are important salt-stress signalling molecules [69]. For example, ROS triggers cytoplasmic calcium to increase during salt stress perception, regulate ion homeostasis and act as second messengers that induce antioxidant defences in herbaceous species [70–72] and trees [73, 74]. Concerning gene expression linked to the antioxidant status of cells, we observed an increase of transcripts encoding superoxide dismutase, a key enzyme involved in the abatement of superoxide anions under salt [10], even though no changes in the activity of this enzyme were observed [30]. Transcripts encoding L-ascorbate peroxidase 6 isoform X2 were down-regulated by salt treatments according to the reduction of ascorbate peroxidase activity [30].

As a final result of the weak activation of the antioxidant system, a strong increase in malondialdehyde by-products (an index of lipid peroxidation) was observed in S plants [30] and some membrane repairing mechanisms were enhanced, as confirmed by the over-expression of transcripts encoding 3-oxoacyl-[acyl-carrier-] reductase, acyl-[acyl-carrier-] desaturase chloroplastic-like and oxalate-ligase-like carboxylesterase 5.

### Differential gene expression under ozone treatment

As in response to salinity, calcium or protein kinases are known to play important roles in O_3_ responses [75]. It has been observed that calcium channels were activated in response to O_3_, and increased cytosolic calcium induced ozone-responsive genes [76]. In our experiments, no genes encoding the Ca^2+^ channel were differentially expressed following O_3_ exposure. On the other hand, other Ca^2+^ related transcripts were among the most over-expressed in O_3_ plants as for example those encoding calmodulins that are sensor relay proteins unique to plants and are involved in many stress responses [77].

The analysis of major hormone pathways revealed no changes of transcript accumulation concerning ABA signalling following O_3_ treatment. Differential regulation of some genes involved in ethylene biosynthesis or in the ethylene network was observed. Also, two auxin-induced transcripts were over-expressed, suggesting the possible involvement of auxin-mediated factors in the oak response to O_3_ treatment. These data confirm complex interactions between hormones in tree responses to O_3_ exposure [78, 79].

Ozonated plants had similar net photosynthetic rates when compared to control plants, suggesting no damage to photosystems or to the Calvin-Benson cycle components [30]. However, under-expression of transcripts involved in the photosynthetic process was observed. One encoded a chlorophyll *a*-*b* binding protein involved in photosystem II (CP26), which mediates the distribution of excitation energy between photosystems II and I [80] preventing over-excitation of thylakoid membranes. In addition, the down-regulation of several gene sequences encoding Rubisco interacting proteins (such as Rubisco large subunit-binding protein, subunit alpha and beta of chloroplastic chaperonin 60,) was observed.

O_3_ exposure induces the accumulation of ROS with their dual role, i.e., toxic compounds and signal molecules [81]. Several transcripts over-expressed by an O_3_ pulse are related to ROS and Halliwell-Asada cycle, such as catalase isozyme, L-ascorbate peroxidase, and 1-aminocyclopropane-1-carboxylate oxidase, an enzyme regulated by glutathione to induce ethylene synthesis during stress [82]. All these enzymes are involved in maintenance of a high antioxidant capacity to scavenge ROS [83]. These results partially agree with the biochemical study by Guidi et al. (2017) in which activities of antioxidant enzymes such as superoxide dismutase and catalase slightly decreased while ascorbate peroxidase and glutathione reductase increased after O_3_ treatment [30].

Other highly up-regulated gene sequences encode membrane receptors such as leucine-rich and cysteine-rich receptor protein kinases. These receptors are likely involved in signalling/sensing the ROS production triggered in the apoplast [13, 84]. Some of these receptors are also implicated in cell wall integrity maintenance because they are capable of detecting cell wall fragments or changes in cell wall composition/structure [85]. *Q. ilex* is characterised by a great thickness of the cell wall [50, 64], which is an early target of O_3_ [86]. Interestingly, several transcripts related to cell wall sugar and protein turn-over were over-expressed after O_3_ treatment, suggesting cell wall structural modifications and consequently cell wall thickening [87].

Guidi et al. (2017) showed O_3_ treatment increased malondialdehyde by-product accumulation, indicating lipid peroxidation [30]. Gene expression analyses showed the accumulation of transcripts related to lipid metabolism encoding glycerophosphodiester phosphodiesterase (GDPDL3), palmitoyl-acyl carrier, omega-6 fatty acid endoplasmic reticulum isozyme 2-like, squalene epoxidase1, confirming lipid metabolism alterations. In addition, squalene epoxidase is involved in sterol biosynthesis, a pathway strictly linked to ROS production, playing an essential role in the localisation of NADPH oxidases required for regulation of ROS under stress conditions [88].

A number of gene sequences up-regulated by O_3_ in our experiments (e.g., salt-activated ATPases, calcineurin B, disease resistance genes such as those encoding TMV resistance N-like, disease resistance RPP8 2 and RGA3, phosphatidylinositol:ceramide inositolphosphotransferase) are also known to be activated in other abiotic or biotic stress conditions, suggesting common response pathways among different stresses [27] and confirming accumulation of defence-related transcripts in O_3_-exposed plants [89].

### Differential gene expression under combined salt and ozone treatment

Comparing responses to combined and single treatments, many differentially expressed transcripts detected in individual O_3_ and/or S treated plants were also differentially expressed after the combined S + O_3_ treatment, suggesting common stress molecular responses, for example those related to production, decoding, detoxification of ROS [90], and calcium-, phytohormone- and protein kinase-signalling pathways, as observed in others species treated with a combination of stresses [27]. For most of these shared transcripts, the fold change was similar between individual and combined stress and, when two single treatments showed a contrasting expression pattern, the final response to combined stress was often determined by the more severe condition (salt stress), as in other studies on plants subjected to multiple stress combinations [27]. Examples are transcripts encoding subtilisin-like proteases that, after S or S + O_3_ treatment, were under-expressed but were up-regulated after individual O_3_ treatment_._ Such proteins were related to plant-pathogen reactions and have recently been associated with a number of aspects of the plant life cycle, including cell wall modification, processing of peptide signals, and biotic and abiotic stress signalling [91]. However, our results showed also opposite expression pattern between individual and combined stress of shared transcripts, suggesting new regulation factors take place in response to combined treatment.

In plants under S + O_3_ stress, a high number of “unique” (i.e., specific of the combined stress) differentially expressed transcripts were found. Many of these transcripts are members of the same gene family; for example, different transcripts encoding members of the NAC-domain transcription factor family that were differentially regulated when comparing S + O_3_ to S or O_3_ plants. It is known that this gene family is strongly regulated by different stresses [92]. Moreover, a number of different gene sequences encoding F-box family proteins, known to have a role in plant development, hormone signalling and defence pathways were specifically regulated by the combined treatment [93].

Results indicate that genes specific of the combined stress are mainly involved in stress signalling, osmotic adjustments, ROS scavenging and signalling, primary metabolism and cell wall remodelling and hereafter will be discussed.

Although some transcripts related to ethylene biosynthesis (aminocyclopropane carboxylate oxidase and S-adenosyl-methionine decarboxylase) were activated in S, S + O_3_ and O_3_ plants, the combination of the two stresses promoted the transcription of specific isoforms of these sequences compared to that activated by O_3_. Similarly, a transcript encoding a SKI interacting-like, involved in modulation of stress resistance through transcriptional regulation of salt stress-related genes [94], was not regulated in S plants but was regulated only after the combined stress.

Concerning osmotic adjustment, it is known that high levels of sucrose help plants to overcome salt stress conditions [25] and trehalose is considered both as an excellent candidate to preserve the lipid bilayer integrity and as an osmoprotectant [95]. One transcript encoding a putative sucrose-phosphate synthase was over-expressed in S + O_3_ plants, however we found down-regulation of a gene sequence encoding an alpha-trehalose-phosphate synthase, an enzyme involved in trehalose biosynthesis. On the other hand, a transcript encoding a putative inositol transporter 2 and a D-xylose-proton symporter were strongly over-expressed, indicating sugar-specific transcriptional alterations of gene sequences related to sugar metabolism occur only in combined stress.

Interestingly, a number of genes that are commonly described as responding to O_3_ [81] or to salt treatments were up-regulated only when these two stresses were applied simultaneously, while in saplings treated only with a single stress, these genes were not regulated. Examples are given by many sequences related to ROS detoxification and related to mitogen-activated kinases presumably involved in ROS sensing, [96]. Also, the regulation of ROS production and detoxification of plants under ozone alone or in combination with salinity differed at the transcription level. While transcripts encoding one L-ascorbate peroxidase cytosolic were over-expressed both after S and S + O_3_ treatment, transcripts encoding one isoform of Mn-SOD, two catalase isozymes, and three members of glutathione transferases gene family were specifically activated only by the combined S + O_3_ treatment. These data partially confirm the biochemical data, which showed no interactive effects of O_3_ and salinity on the activity of SOD and catalase and a strong increase of ascorbate peroxidase activity [30].

Transcripts related to photosystem II, such as photosystem II 22 kDa chloroplastic, were under-expressed after the combined treatment, as already observed after salt treatment. However, the combined treatment led to down-regulation of a higher number of gene sequences encoding photosystem II proteins (for example, photosystem II Z and photosystem II M) compared to the salt treatment alone.

As observed in the leaves of S plants, transcripts encoding mitochondrial, vacuolar and chloroplastic ATP synthase were over-expressed in the leaves of S + O_3_ plants. However, two gene sequences encoding a transketolase-chloroplastic and a glyoxylate hydroxypyruvate reductase, two key enzymes involved in plant carbon metabolism and photorespiration, respectively, were over-expressed only in S + O_3_ leaves, suggesting transcriptional modifications for stimulating the regeneration phase of the Calvin-Benson cycle or the photorespiratory process, as observed in other stress conditions [97, 98].

Concerning genes related to cell wall remodelling, the combined treatment regulated a higher number of gene sequences than O_3_ or the salt treatment separately. Among these, a transcript encoding a shikimate O-hydroxycinnamoyl transferase, which is related to lignin biosynthesis [99], was under-expressed only in the combined treatment. A transcript related to inositol oxygenase, known to be involved in the biosynthesis of nucleotide sugar precursors for cell wall matrix polysaccharides [100], resulted in being strongly over-expressed only in S + O_3_ plants. Interestingly, concerning the pectinesterase gene family, both O_3_ and S + O_3_ plants showed a strong increment of pectinesterase 63 transcripts, whereas S + O_3_ plants showed a decrement of pectinesterase 2 and 11 transcripts, indicating a stress-specific fine tuning of transcription of genes belonging to this family.

Taken together, these data suggest the response to a combination of stresses cannot be predicted easily and cannot be directly extrapolated from the response of plants to each of the different stresses applied individually.

## Conclusions

In our experiments, we studied *Q. ilex*, an urban-adapted plant that is very common in cities at different latitudes and is adapted to environments very different from natural ones. We analysed changes in its transcriptome profile by mimicking two realistic stressful conditions that may occur regularly in a Mediterranean urban environment.

Here, we produced for the first time a reference transcriptome for *Q. ilex* subjected to salt, ozone and their combination.

Overall, the transcriptome analysis unveils that salinity dramatically changed the profile of gene expression, whereas the impact of ozone was less severe. However, the short O_3_ pulse had a very strong effect on changing the *Q. ilex* transcriptome, when it was combined with the salt treatment. Although responses to combined and individual stresses shared a number of regulated genes, several differences were observed in their expression level.

Moreover, new specific transcripts were detected under combined stress conditions in respect to individual treatments.

Our data indicate expression changes in response to combined stress is a unique adaptation strategy tailored for stress combination, which is perceived by the plant as a new stress, leading to major remodelling of gene expression [27, 101].

In conclusion, the transcriptome and expression data reported here are a reliable dataset that will be useful for future studies aiming to define gene expression in urban-adapted plants to be conducted in vivo, i.e., in plants really living in urban environments, for unravelling the complex biochemical and physiological patterns allowing plants to adapt to this extreme and unnatural environment.

## Additional files

Additional file 1: **Table S1.** Blastn comparison between de novo assembly transcriptome (*Q. ilex*) and OCV3_91K reference transcriptome (*Q. robur* and *Q. petraea*, Lesur et al. 2015) [35]. (XLSX 6828 kb)

Additional file 2: **Table S2.** Differentially expressed genes in response to salt (based on log fold change, with FDR < 0.05) compared to control plants. (XLSX 150 kb)

Additional file 3: **Table S3.** Differentially expressed genes in response to ozone (based on absolute log fold change, with FDR < 0.05) compared to control plants. (XLSX 30 kb)

Additional file 4: **Table S4.** Differentially expressed genes in response to salt plus ozone (based on log fold change, with FDR < 0.05) compared to control plants. (XLSX 187 kb)
